# Assessing the Changes of Mumps Characteristics with Different Vaccination Strategies Using Surveillance Data: Importance to Introduce the 2-Dose Schedule in Quzhou of China

**DOI:** 10.1155/2020/8130760

**Published:** 2020-03-27

**Authors:** Chunting Zhou, Wei Song, Zhiying Yin, Sheng Li, Xiaoying Gong, Quanjun Fang, Shuangqing Wang

**Affiliations:** ^1^Women & Children Health Care Hospital of Quzhou, Quzhou, 324000 Zhejiang, China; ^2^Department of Immunization, Quzhou Center for Disease Control and Prevention, Quzhou, 324000 Zhejiang, China

## Abstract

**Background:**

From 2005 to 2016, the prevention and control of mumps in China have undergone three stages of transition. These include the use of MuCV as a self-supported vaccine, the introduction of one-dose MMR to the Expanded Program on Immunization (EPI), and the administration of two-dose MuCV following supplementary immunization activities (SIAs) using MM. Here, using surveillance data, we assessed the epidemiology of mumps during the three stages.

**Methods:**

Children in Quzhou of China born from 2005 to 2016 and registered in the Zhejiang Provincial Immunization Information System (ZJIIS) were included. We analyzed the epidemic data and calculated incidence and MuCV coverage via birth cohorts.

**Results:**

The average incidence of mumps in 2005-2006, 2007-2010, and 2011-2016 was 51.57, 41.02, and 12.53 per 100,000 individuals, respectively. The highest incidence was in children aged 6-14 years from 2005-2016, of which the majority were school students (67.84%). Approximately 90% of the reported outbreaks occurred in school children (primary school/middle school). The seasonal characteristics of mumps were less obvious from 2011 to 2016. The coverage of one-dose MMR in the 2005 birth cohort was 71.38%. For the 2006-2010 birth cohort, the coverage of one-dose MuCV was 96.82% and the coverage of two-dose MuCV was 17.68%. The children born from 2011 to 2016 were only free vaccinated with MMR; the coverage of one-dose MuCV was 99.10%. The mumps incidence in the three birth cohorts significantly declined (*X*^2^ = 805.90, *P* < 0.001 for trend). Except the children less than two years old, the mumps incidence for the children born from 2006 to 2010 was higher than that for the children born from 2011 to 2016.

**Conclusion:**

The mumps incidence significantly declined following the introduction of one-dose MMR. The SIA using MM led to a rapid reduction of mumps cases. Therefore, we recommend a two-dose MuCV routine immunization schedule and improved vaccination coverage.

## 1. Introduction

Mumps is a contagious disease caused by the mumps virus. It typically starts with fever, headache, muscle ache, tiredness, and loss of appetite. The majority of sufferers also develop swelling of the salivary glands [1]. In China, mumps was a national statutory C infectious disease in 1990, and all mumps cases have been mandatorily reported via the National Notifiable Disease Reporting System (NNDRS) since 2004, a web-based computerized reporting system. Reported incidence rates are approximately 22 per 100,000 within the total population but have reached as high as 89.91 per 100,000 in 2009 in one province. The number of reported mumps outbreaks was 436, 327, and 194 for 2008, 2009, and 2010, respectively [2]. Due to the outbreaks and high incidence rates, mumps prevention in China needs to be strengthened and improved.

Mumps vaccine (MuV) is the most effective strategy for mumps protection. In 1990, the China Food and Drug Administration licensed a live, attenuated mumps vaccine that was produced using the S79 vaccine strain, derived through attenuation of the Jeryl Lynn strain used in the U.S.-licensed vaccine [3]. Mumps vaccination was initiated in Quzhou since 1998 using two mumps-containing vaccines (MuCV) including the monovalent mumps vaccine (S79 strain) and the measles–mumps–rubella (MMR) vaccine developed by Merk (Jery1-Lynn vaccine strain). Monovalent mumps vaccines were replaced with the measles–mumps (MM) vaccine (S79 strain) since 2000. However, MuVs were not included in the Expanded Program on Immunization (EPI), meaning parents were forced to pay sums for the MuV. In 2007, domestic MMR (S79 strain) was introduced into the EPI for children born after the 1st January 2006 and replaced the second routine dose of measles vaccine, targeting children aged 18–24 months. Since the first dose of measles-containing vaccine is administered as the measles-rubella (MR) vaccine, the EPI system supports only one-dose mumps vaccination strategy [4]. Though MMR was introduced into the EPI for routine use with high vaccination coverage, over 1,839 mumps cases were reported in 2009 in Quzhou. The majority of outbreaks occurred amongst school-age children [5]. Outbreaks have been reported amongst highly vaccinated populations in numerous countries [6–8]. In September 2010, supplementary immunization activities (SIAs) using MM were performed, targeting children aged 8 months to 4 years of age. The Zhejiang Provincial Immunization Information System (ZJIIS), also known as the immunization registries, is a computerized population-based system containing demographic and vaccination data for all children aged less than 15 years living in the Zhejiang Province since 2004 [9]. We analyzed mumps epidemiology and MuCV coverage of Quzhou using the NNDRS and ZJIIS from 2005 to 2016 in this study.

## 2. Material and Methods

### 2.1. Setting

Quzhou is a medium city of Zhejiang Province in the East of China and includes 2 districts and 4 counties. Based on the annual census data from the Quzhou municipal Bureau of Statistics, its population increased from 2,456,000 in 2005 to 2,649,000 in 2016 (7.86% increase), with an annual birth cohort of approximately 24,000. Quzhou is served by 108 vaccination clinics, which are responsible for vaccinating all children residing in the catchment areas, regardless of whether they were locally born or migrated to Quzhou. Since 2005, all children, including migrant (nonlocally born), were registered in ZJIIS during their first contact with the immunization clinic during which they were administered a unique identification number. The system contains children's demographic information, historical immunization data, and current immunization.

### 2.2. Case and Outbreak Definitions

For surveillance purposes, mumps is defined as a clinically diagnosed illness. According to the diagnostic criteria for mumps approved by the Ministry of Health of China in 2007 [10], we defined a mumps case as a person with acute onset of unilateral or bilateral swelling of the parotid gland or other salivary glands characterized by any of the following, which could not be explained by another more likely diagnosis: (1) fever, headache, weakness, and loss of appetite; (2) orchitis; (3) pancreatitis; (4) encephalitis and/or aseptic meningitis. In Quzhou, a mumps outbreak is defined as the occurrence of ≥10 mumps cases in a community, school, company, or other settings within a seven-day period.

### 2.3. Mumps and Vaccination

Data for patients diagnosed with mumps in Quzhou from 2005 to 2016 were extracted from the NNDRS on 30 March 2017. Data on MuCV vaccination coverage were obtained from the ZJIIS. We defined the birth cohorts from 2005 to 2016 by the number of children enrolled in ZJIIS. We calculated MuCV vaccination coverage using the cumulative number of children who had received the MuCV until the end of each year, divided by the total number of children in the corresponding birth cohort.

### 2.4. Statistical Analyses

We described the epidemic characteristics of mumps occurring from 2005-2016, and MuCV coverage and incidence of mumps by birth cohorts from 2005 to 2016, using the life table method. Data were collected using Microsoft Office Excel (version 2007) and analyzed using SPSS for Windows, version 17.0 (SPSS Inc., USA). Differences amongst incidence periods were calculated using the trend Chi-square test. Differences according to median age were calculated using the Kruskal-Wallis *H* test. All comparisons were 2-tailed, and a *P* value < 0.05 was considered significant.

### 2.5. Ethical Considerations

This study was determined to be exempt from ethical review by the Quzhou CDC institutional review board. Data were anonymous and exported from ZJIIS. Confidentiality without individual identifiers was maintained throughout.

## 3. Results

### 3.1. Mumps Cases and Incidence

The average annual reported incidence of mumps was 28.67 per 100,000 of the population from 2005 to 2016, the overall incidence of which decreased by 86.24% from the maximum 73.91 per 100,000 of the population in 2009 to the minimum 10.17 per 100,000 of the population in 2015. The average incidence of mumps in 2005-2006, 2007-2010, and 2011-2016 was 51.57, 41.02, and 12.53 per 100,000 people, respectively, which declined across the three periods (*X*^2^ = 552.551, *P* < 0.001 for trend).

### 3.2. Demographic Characteristics of Mumps Cases

In the three assessment periods, mumps incidence was highest amongst children aged 6-14 years and lowest amongst adults aged ≥20 years. The incidence amongst children aged less than 2 years did not significantly change (*X*^2^ = 0.062, *P* = 0.969). The incidence amongst children aged 6-14 years declined across the three periods (Table 1).

**Figure 1:**
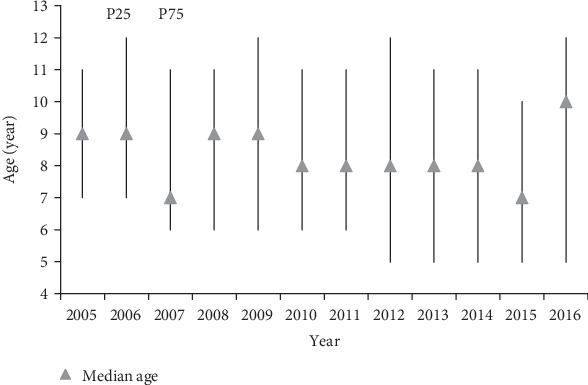
Median age and interquartile range (IQR) of mumps cases from 2005 to 2016.

The median age of mumps cases was almost nine from 2005 to 2009, eight from 2010 to 2014, seven in 2015, and ten in 2016, which significantly differed (*X*^2^ = 138.001, *P* < 0.001). The interquartile range (IQR) changed from five to seven and ranged from 5 years to 12 years (Figure 1).

Of the 8,525 cases, 5,214 (61.16%) were male. The majority of cases (67.84%) were school students, and 1,646 (19.31%) of cases were childcare children in kindergartens. The proportion of students steadily decreased from 79.94% in 2005 to 64.18% in 2016, and the proportion in childcare gradually increased from 13.34% in 2005 to 22.09% in 2016.

### 3.3. Seasonal Characteristics of Mumps Cases

**Figure 2:**
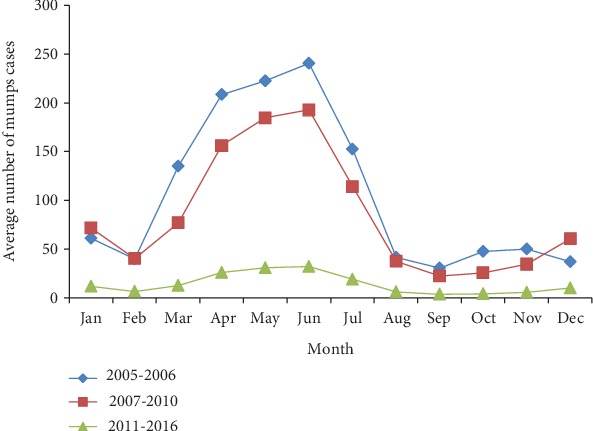
Average number of mumps cases in Quzhou per month during the three assessment periods.

Mumps cases were reported throughout all 12 months of the year. No obvious seasonal patterns were observed despite its reported seasonal occurrence. Most mumps cases occurred between March and July, with small peaks also occurring in December and January in 2005-2006 and in 2007-2010. With the decreased number of mumps cases in 2011–2016, the two epidemic peaks were less obvious than those observed from the other study periods (Figure 2).

### 3.4. Outbreaks

From 2005 to 2016, a total of 21 outbreaks with 919 cases were reported. Approximately 90% of the reported outbreaks occurred in school children (primary school/middle school), which accounted for the majority (approximately 95%) of outbreak-related cases. Since 2008, no outbreaks in kindergartens had been reported. Since 2010, no outbreak in primary schools had been reported. From 2011 to 2016, no mumps outbreaks were reported (Table 2).

### 3.5. Incidence of Mumps Cases by Birth Cohort

The children born in 2005 vaccinated MuCV must pay sums, the coverage of one-dose MuCV was 71.38%, and the incidence of mumps was 138.56 per 100,000 person-years. The children born from 2006 to 2010 were free vaccinated with MMR and boost immunity using MM, and the coverage of one-dose MuCV and two-dose MuCV was 96.82% and 17.68%, respectively. The children born from 2011 to 2016 were only free vaccinated with MMR, and the coverage of one-dose MuCV was 99.10%. The incidence of mumps in the three birth cohorts significantly declined (*X*^2^ = 805.90, *P* < 0.001 for trend) (Table 3).

**Figure 3:**
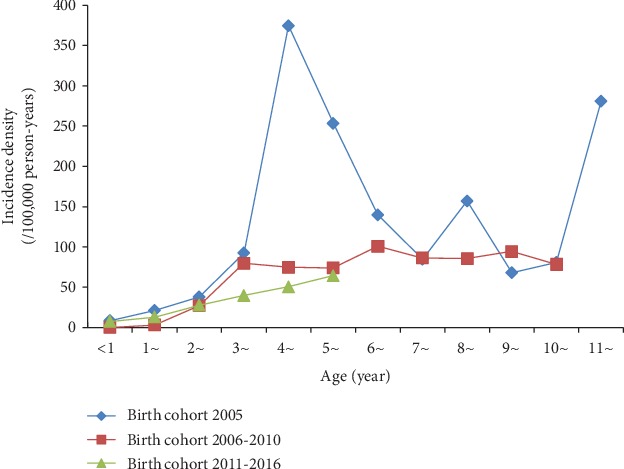
Incidence of mumps amongst different birth cohorts by age.

The incidence of mumps in children born in 2005 had two peaks: the one was 4–5 years old and the other was 11–12 years old. Except the children less than two years old, the incidence of mumps for children born from 2006 to 2010 was higher than that for children born from 2011 to 2016 by age (Figure 3).

## 4. Discussion

Mumps is a vaccine-preventable disease. Since the prevaccination era, a 99% decrease in mumps cases has been observed in the United States [1]. In 2015, amongst the 194 World Health Organization (WHO) countries, 121 (62%) had incorporated MuV into their national immunization program, the majority of which used the MMR vaccine [11]. From 2005 to 2016, the prevention and control of mumps has gone through three periods in Quzhou. The first occurred in 2005-2006 in which MuCV was used as a self-supported vaccine. The second was from 2007 to 2010 in which one-dose MMR was introduced into the EPI for children aged 18–24 months. The third was from 2011 to 2016 in which some children were administered two-dose MuCV, including one-dose MM of SIAs and one-dose MMR of EPI. Our studies have shown that 8,525 mumps cases with an average annual incidence of 28.67 per 100,000 of the population were reported in Quzhou in 2005–2016. This was higher than that of Beijing and Jiangsu province [12, 13]. The average mumps incidence from 2007-2010 modestly decreased compared to 2005-2006, due to the short timeframe of MMR vaccine introduction and the susceptible population with a low mumps vaccination coverage. However, these values drastically declined from 2011 to 2016 (*X*^2^ = 552.551, *P* < 0.001). A single dose of the MMR vaccine used in the UK, which contains the Jeryl Lynn mumps strain, has been reported to confer between 61 and 91% protection [14].

Mumps is a common childhood infection in unimmunized individuals, but in highly vaccinated populations, the disease affects mainly adolescents and young adults [15–18]. From 2005 to 2016, the age distributions for mumps cases also deviated. In children aged less than 2 years and adults aged ≥20 years, no obvious changes were evident since these did not represent the susceptible or target population of EPI. However, the number of mumps cases amongst adults increased in some provinces [19]. From 2005-2010, only some of the children aged 2–5 years were the target population of EPI, and thus, the incidence was volatile. The inclusion of all children as the target EPI population since 2011 has led to the incidence significantly declining. The susceptible population of mumps infection was mainly comprised of children aged 6-14 years, who had not received the mumps vaccination from 2005-2010. In 2016, the incidence amongst those aged 15–19 years significantly increased, suggesting that mumps is not simply a childhood disease. The most afflicted age groups were teenagers, adolescents, and young adults, similar to those previously reported [20]. A Korean study also identified the need to strengthen surveillance in adolescents, in addition to younger aged children [21].

According to the median age of mumps from 2005-2016, the highest incidence rates occurred in children aged 6–14 years. The other birth cohorts excluding 2006 and 2008 had low mumps vaccination coverage as the MuCV was only introduced into the EPI for children born after 1 January 2006, and the time after vaccination with MuCV was assessed in the future. The issue of the growing risk of developing mumps at increasing postvaccination has been addressed [22, 23]. Studies suggested that humoral immunity induced by live vaccines at childhood, including measles and mumps antibodies, does not provide lifelong immunity but can rapidly decline to extremely low levels by adolescence and young adulthood. This situation is particularly apparent in populations lacking boosters derived from natural infections [24]. During the epidemic season, the two epidemic peaks were less obvious in 2011–2016 than in the other two periods.

Of all the mumps cases assessed, 61.16% were male, and 67.84% were students in schools. These values may be attributable to sex-based immune responses and hormonal influences in addition to genetic and epigenetic factors [25, 26]. The accumulation of susceptible young persons who are brought together in high-density settings can lead to high levels of infection and an increased risk of exposure [27, 28]. Mumps outbreaks were recently reported in the US and in Europe, both with high MMR vaccine coverage [27, 29]. From 2005 to 2016, 21 mumps outbreaks were reported, and primary school students accounted for 90.21% of the outbreak-related cases. No mumps outbreaks were reported from 2011 to 2016. Both factors are consistent with the observation that the mumps outbreak primarily affected students [15, 30–32]. Compared to the 2005-2006 and 2007-2010 periods, the mumps epidemiological characteristics remained unchanged from 2011-2016. There were three possibilities for the changes. Firstly, the susceptible populations decreased due to the mumps infection. Secondly, children with MuCV vaccination had accumulated. Thirdly, the SIAs using MM could reduce the number of individuals who failed immunization with MuCV and increased the doses of MuCV immunizations. Studies also revealed the importance of waning immunity and the assessment of the time since vaccination [33]. Thus, continuing epidemic surveillance for mumps is necessary to understand whether the inclusion of MuCV will break the natural epidemic cycle of mumps in Quzhou and help maintain low incidence levels.

We also observed that the incidence of mumps cases assessed by birth cohort was connected to the coverage of MuCV and the doses of MuCV administered. The coverage of one-dose MuCV in the 2005 birth cohort was 71.38%. The coverage of one-dose MuCV and two-dose MuCV in 2006-2010 birth cohorts was 96.82% and 17.68%, respectively. The coverage of one-dose MuCV in 2011-2016 birth cohorts was 99.10%. The incidence of mumps in the three birth cohorts significantly declined (*X*^2^ = 805.90, *P* < 0.001 for trend). The WHO reported that the MuCV coverage should reach 90% to prevent a mumps outbreak [34]. Studies performed in a French cohort concluded that the effectiveness of the MuV decreases with time and therefore proposed the introduction of a targeted third dose in an outbreak setting, for individuals whose last dose was longer than 10 years earlier [35]. It has been shown that valid and invalid vaccination rates influence the spread of mumps, but vaccine coverage and the transition to two doses of MMR vaccine were made freely available in China [36]. Our analysis concludes that the preventive effects of one-dose MuCV above a 90% coverage were limited and that a routine immunization schedule of two-dose MuCV for children is urgently required.

There were several limitations to this study: (1) all mumps cases were clinically diagnosed without laboratory confirmation. Mumps virus infection can result in symptomatic or asymptomatic infections [37], and the estimated 20–30% of asymptomatic cases were not possible to identify [3]. Mumps incidence in this study may therefore be underestimated or overestimated. (2) The immunization status of each mumps case was unknown, and we were unable to calculate the effectiveness of various vaccine doses as the NNDRS and ZJIIS data were disconnected. (3) MuCV vaccination coverage may have been underestimated since children with prior mumps infections were not excluded from the study cohort.

In conclusion, the coverage of one-dose MMR has reached approximately 90% since its introduction into the EPI in Quzhou, and the incidence of mumps in target children has significantly declined. Due to the short time of EPI initiation, mumps-susceptible individuals with no MuCV immunization history increased the mumps incidence, and the SIAs using MuCV can improve mumps antibody levels in the target population over a short timeframe, which led to a rapid reduction in mumps cases. A further decrease in mumps incidence could be achieved through the introduction of two doses of MuCV and by improving 2-dose MuCV vaccination coverage. However, determining the optimal age and adjustment of the schedule will require a further consideration of laboratory and serological survey results [20].

## Figures and Tables

**Table 1:** The incidence of mumps amongst age groups in the three assessment periods.

Age group	2005-2006		2007-2010		2011-2016		*X* ^2^	*P*
	No.	Incidence (1/100,000)	No.	Incidence (1/100,000)	No.	Incidence (1/100,000)		
<2 y	12	12.63	24	12.30	38	11.74	0.062	0.969
2–5 y	289	131.92	700	179.72	506	84.97	171.775	<0.001
6–14 y	1947	352.82	2932	281.21	1141	76.50	2035.562	<0.001
15–19 y	144	39.78	220	30.96	90	10.83	112.646	<0.001
≥20 y	141	3.83	197	2.60	144	1.19	110.152	<0.001

**Table 2:** No. of mumps outbreaks and outbreak-related cases from 2005 to 2016.

Period	Kindergarten		Primary school		Middle school		Total	
	No. of outbreaks	No. of cases	No. of outbreaks	No. of cases	No. of outbreaks	No. of cases	No. of outbreaks	No. of cases
2005-2006	1	12	7	346	0	0	8	358
2007-2010	1	35	10	483	2	43	13	561
2011-2016	0	0	0	0	0	0	0	0
Total	2	47	17	829	2	43	21	919

**Table 3:** Incidence of mumps and coverage of MuCV in different birth cohorts.

Birth cohort	No. of children	Coverage of one-dose MuCV (%)	Coverage of two-dose MuCV (%)	No. of cases	Cumulative exposure (person-year)	Incidence (/100,000 person-years)
2005	23824	71.38	0	377	272079.5	138.56
2006-2010	123157	96.82	17.68	804	1035725.5	77.63
2011-2016	149827	99.10	0	201	446128	45.05

## Data Availability

The data used to support the findings of this study are available from the corresponding author by email (yzy1815@sohu.com).
